# Author Correction: Machine learning-led semi-automated medium optimization reveals salt as key for flaviolin production in *Pseudomonas putida*

**DOI:** 10.1038/s42003-025-08141-5

**Published:** 2025-05-13

**Authors:** Apostolos Zournas, Matthew R. Incha, Tijana Radivojevic, Vincent Blay, Jose Manuel Martí, Zak Costello, Matthias Schmidt, Tan Chung, Mitchell G. Thompson, Allison Pearson, Patrick C. Kinnunen, Thomas Eng, Christopher E. Lawson, Stephen Tan, Tadeusz Ogorzalek, Nurgul Kaplan, Mark Forrer, Tyler Backman, Aindrila Mukhopadhyay, Nathan J. Hillson, Jay D. Keasling, Hector Garcia Martin

**Affiliations:** 1 Biological Systems and Engineering Division, Lawrence Berkeley National Laboratory, Berkeley, CA 94720 USA; 2 Department of Energy Agile BioFoundry, Emeryville, CA 94608 USA; 3 Joint BioEnergy Institute, Emeryville, CA 94608 USA; 4 Department of Bioengineering, University of California, Berkeley, CA 94720 USA; 5 Biomaterials and Biomanufacturing, Sandia National Laboratories, Livermore, CA 94550 USA; 6 Department of Chemical & Biomolecular Engineering, University of California, Berkeley, CA 94720 USA; 7 BCAM, Basque Center for Applied Mathematics, Bilbao, 48009 Spain

**Keywords:** Industrial microbiology, Machine learning

Correction to: *Communications Biology* 10.1038/s42003-025-08039-2, published online 18 April 2025

In the version of the article initially published, Matthias Schmidt’s surname appeared incorrectly (as Schimdt) and has now been corrected in the HTML and PDF versions of the article.

