# Supplementary material for: Machine learning-led semi-automated medium optimization reveals salt as key for flaviolin production in Pseudomonas putida
Source: Commun Biol. 2025 Apr 18;8:630. doi: 10.1038/s42003-025-08039-2 (PMC12008372; doi:10.1038/s42003-025-08039-2)
Supplement: Supplementary file 2 — Supplementary Material [file 42003_2025_8039_MOESM2_ESM.pdf]

Supplementary material for:

Machine learning-led semi-automated medium optimization reveals salt as key for flaviolin production in *Pseudomonas putida*

Apostolos Zournas[1,2,3]*, Matthew R. Incha[1,2,3]*, Tijana Radivojevic[1,2,3], Vincent Blay[1,3], Jose Manuel Martí[1,2,3], Zak Costello[1,2,3], Matthias Schimdt[1,3], Tan Chung[1,3,4], Mitchell G. Thompson[1,3], Allison Pearson[1,3], Patrick C. Kinnunen[1,2,3], Thomas Eng[1,3], Christopher E. Lawson[1,3], Stephen Tan[1,2,3], Tadeusz Ogorzalek[1,2,3], Nurgul Kaplan[1,2,3], Mark Forrer[2,3,5], Tyler Backman[1,3], Aindrila Mukhopadhyay[1,3], Nathan J. Hillson[1,2,3], Jay D. Keasling[1,3,4,6], Hector Garcia Martin[1,2,3,7,†]

[1] Biological Systems and Engineering Division, Lawrence Berkeley National Laboratory, Berkeley, CA 94720, USA
[2] Department of Energy Agile BioFoundry, Emeryville, CA 94608, USA
[3] Joint BioEnergy Institute, Emeryville, CA 94608, USA
[4] Department of Bioengineering, University of California, Berkeley, Berkeley, California, 94720, USA
[5] Biomaterials and Biomanufacturing, Sandia National Laboratories, Livermore, California 94550, USA
[6] Department of Chemical & Biomolecular Engineering, University of California, Berkeley, Berkeley, California, 94720, USA
[7] BCAM, Basque Center for Applied Mathematics, Bilbao 48009, Spain.

* These authors contributed equally
[†] Correspondence: hgmartin@lbl.gov

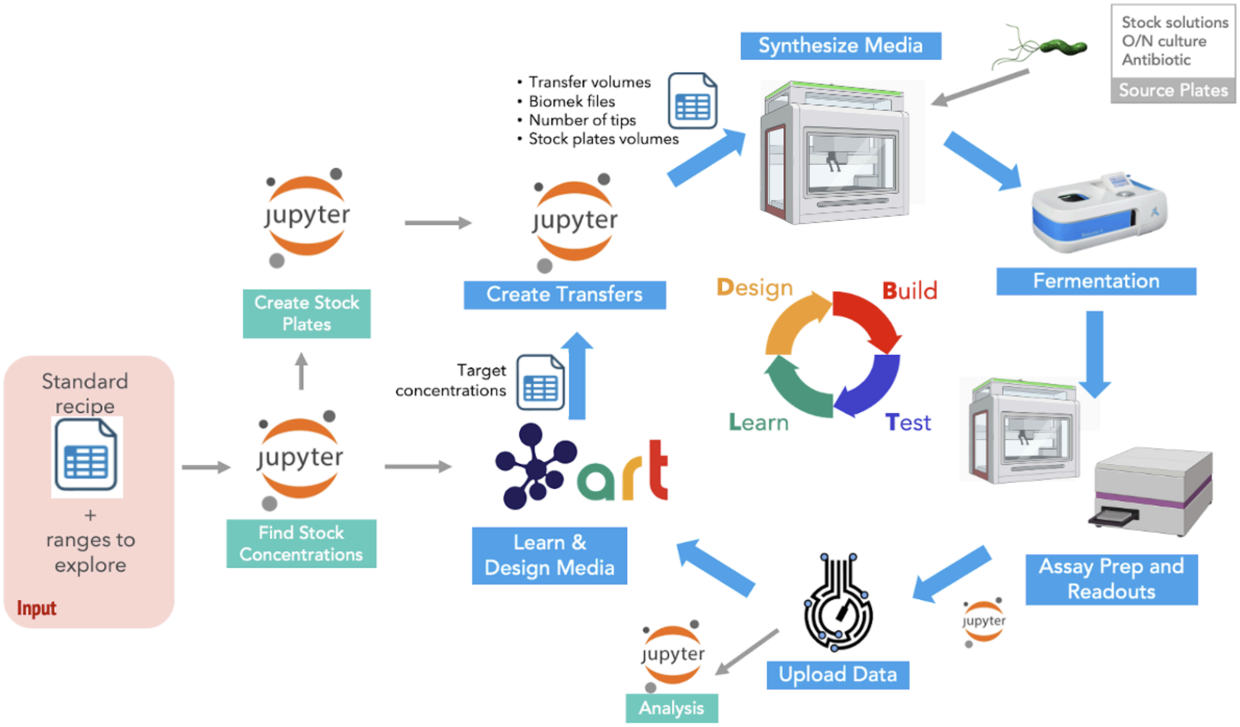

Figure S1: More detailed version of our semi-automated pipeline shown in Fig. 2. The semi-automated pipeline requires the base media composition (standard recipe) and the ranges that will be explored for each media component. Based on these, the media compiler library generates the required stock concentrations as well as the templates for creating the stock plates used in liquid handling. **(D**esign**):** ART suggests media designs within these bounds either in a DoE mode for the first DBTL cycles or in an active learning mode for the following cycles. **(B**uild**):** once the design step is completed using ART, the desired concentrations for each media component are stored in comma-separated values (.csv) format. These concentrations are then read by the media compiler library to generate the required stock plates and instructions for the liquid handler, and we use the Biomek NX-S8 liquid handler to mix the media. **(T**est**):** The inoculated media is then moved to the Biolector for a 48h cultivation, and Abs_{340} is measured in the supernatant post cultivation through the Spectramax M2 microplate reader. The data and metadata containing the media design description is then uploaded to the Experimental Data Depot (EDD). **(L**earn**):** The data is fetched from the EDD and data cleaning and visualization is performed. The newly acquired data is concatenated with the data from previous DBTL cycles and used to train ART, after which the design step is repeated in active learning mode.

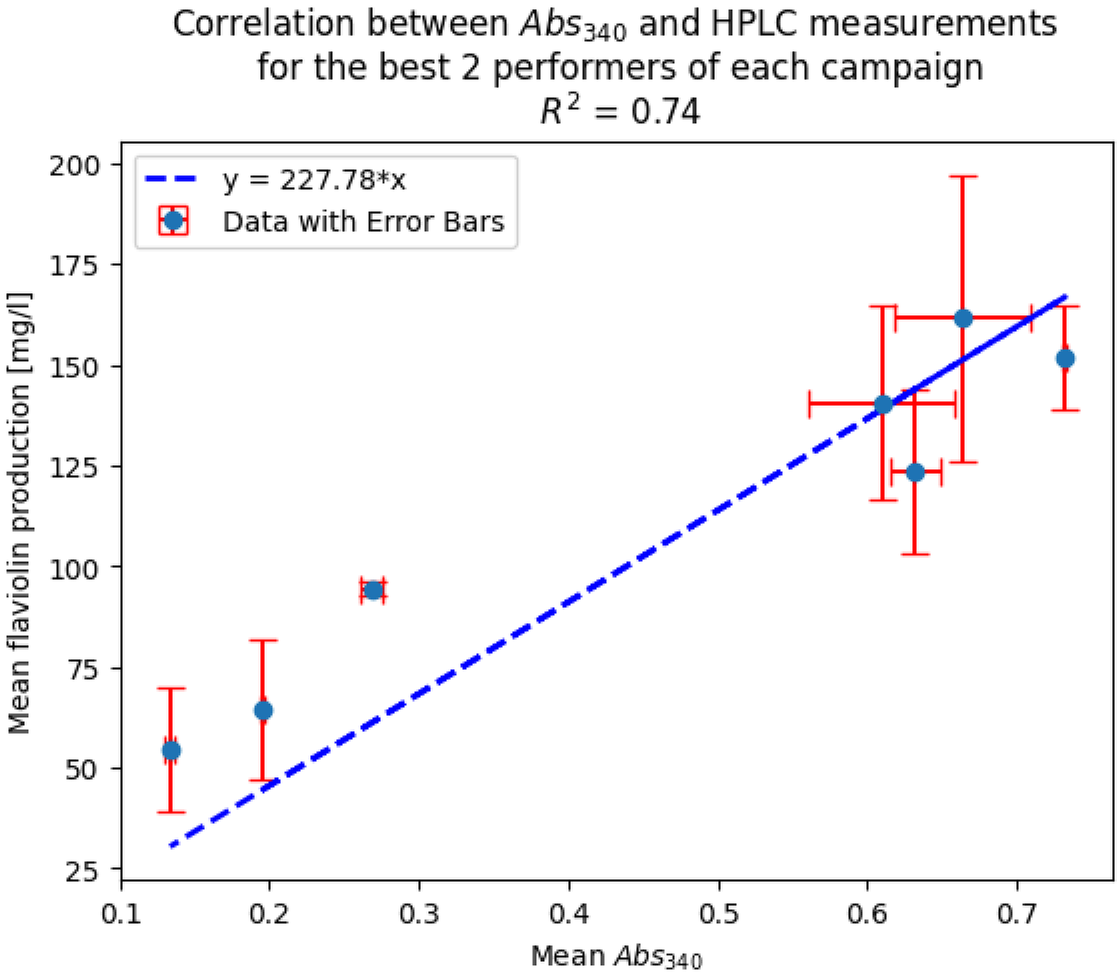

Figure S2: The proxy for flaviolin quantification (Abs_{340}) correlates well with direct HPLC measurements of flaviolin. We took the best two performers from each campaign (Figs. 4 and S4) and directly quantified the flaviolin concentration through High Pressure Liquid Chromatography (HPLC). Error bars indicate the standard deviation from three biological replicates. A coefficient of determination of R^2 = 0.74 indicates that the changes of Abs_{340} explain 74% of the variance in HPLC measurements of flaviolin.

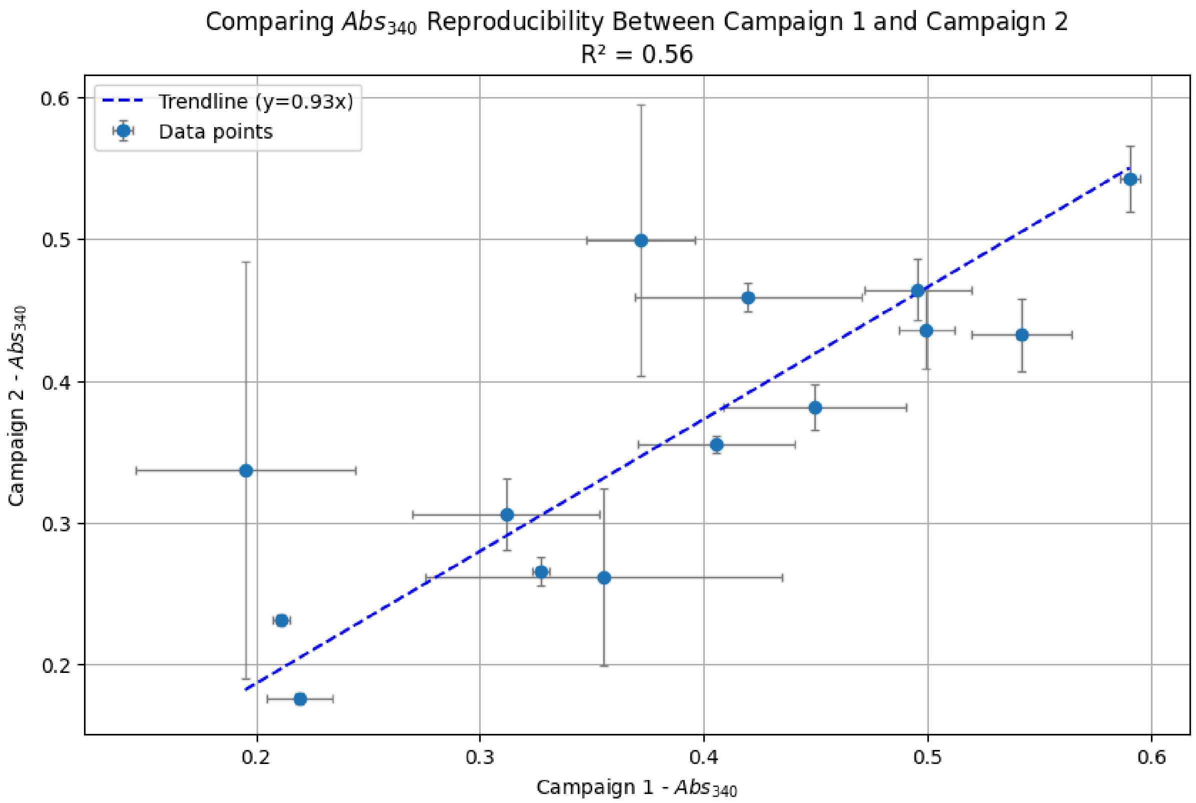

Figure S3: The semi-automated experimental pipeline provides experimental reproducibility for processes one year apart by different users. The same samples, generated by Latin Hypercube Sampling, were tested at the end of Campaign 1 and Campaign 2, carried over one year apart and by different users. The measurements were highly correlated, and a linear regression between the 2 generated a correlation coefficient of 0.93 and coefficient of determination of 0.56, confirming reproducibility between runs. This reproducibility is remarkable since the measurements were taken one year apart, using different initial stock solutions (Fig. S1) and experiments were performed by different operators. The error bars indicate the standard deviation from three biological replicates.

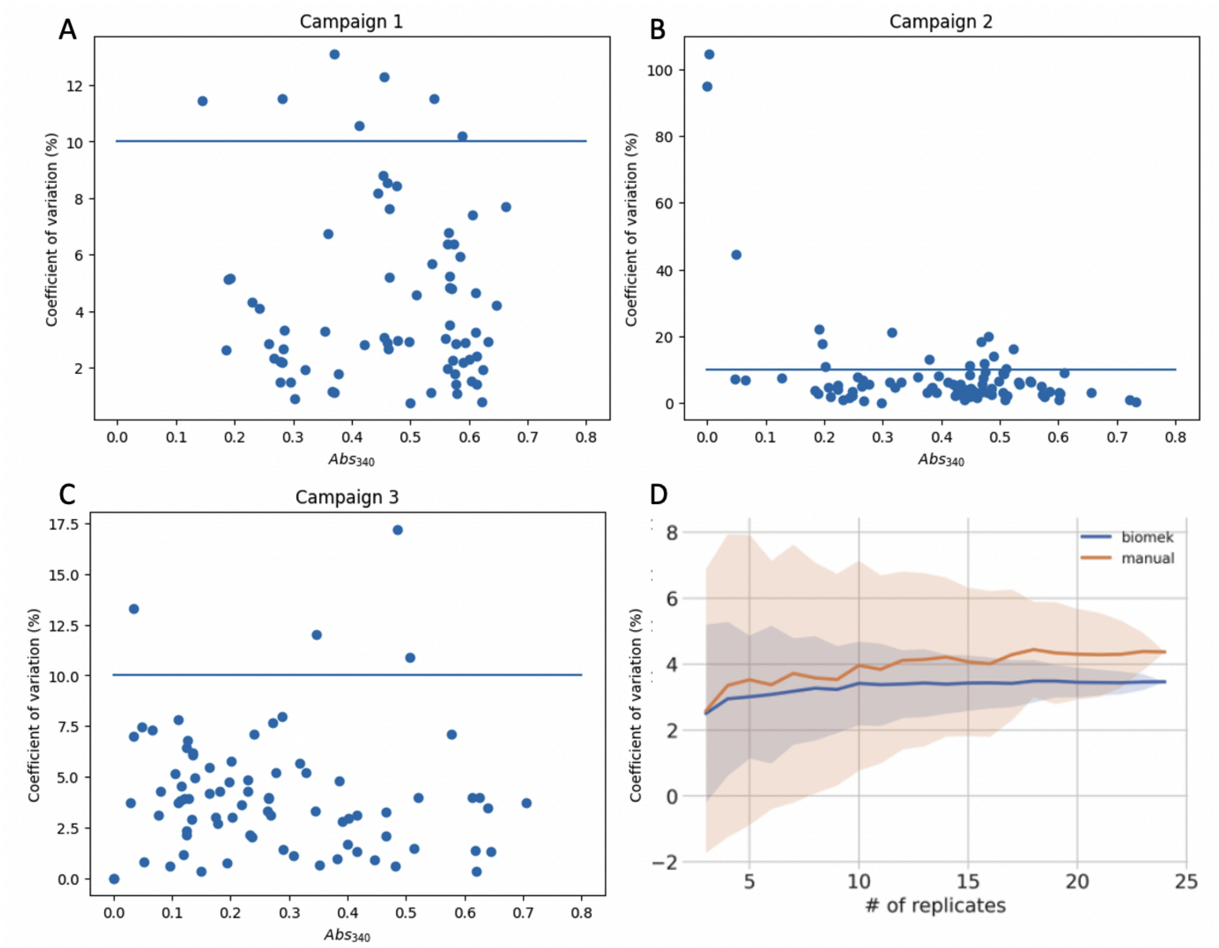

Figure S4: The semi-automated pipeline generates highly reproducible results within a cycle. (A-C) The response coefficient of variation (standard deviation over mean) for most media designs was usually lower than 10% in all campaigns, particularly in media designs generating high Abs_{340} (flaviolin production proxy). **(D)** By using a full biolector plate to test replicates, we observed that the liquid handler produced significantly higher reproducibility than manually built media measured, as measured by a lower coefficient of variation with the same number of replicates (D).

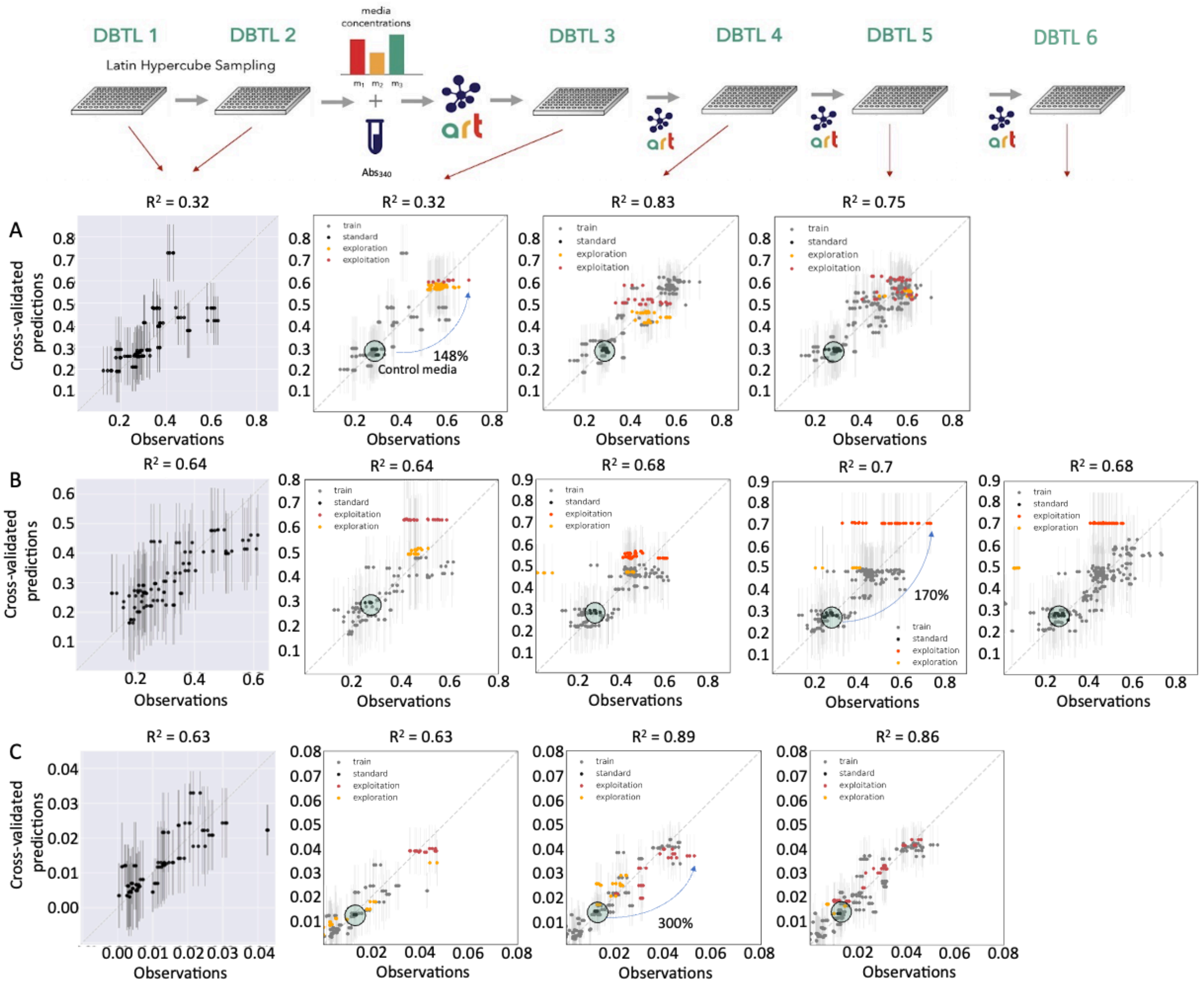

Figure S5: The semi-automated active learning process results in significant improvements in flaviolin titer (C1,C2) and yield (C3) in all three campaigns executed. Each process constituted a campaign, which started with two DBTL cycles using a Design-of-Experiment (DoE) approach to explore as much phase space as possible, and was followed by 3-4 DBTL cycles using machine learning to guide the actual active learning process in which the data from previous cycles was used to train ART and produce recommendations for the next cycle (**Fig. 2**). We executed three campaigns: C1, C2, and C3, and each DBTL cycle included a control of the initial media in triplicate or quadruplicate to ensure reproducibility. Campaign 1 (C1, Fig. 4) used four replicates in DBTL1-2, providing 11 media designs (instances) per cycle. In the following cycles (DBTL 3-5), three replicates were used, providing 15 media designs (instances) per cycle, eventually increasing titer by 148%. Campaign 2 (C2) used three replicates in each cycle, eventually increasing titer by 170%. Campaign 3 (C3) used three replicates and focused on improving yield, eventually increasing it by 300%. The coefficients of variation (standard deviation over mean) for the response (flaviolin titer for C1 and C2, yield for C3) were 0.067, 0.079 and 0.081 (<10%), respectively, demonstrating the repeatability of the pipeline. Predictive power was quantified through the coefficient of determination, R^2, a more stringent measure than correlation coefficients.

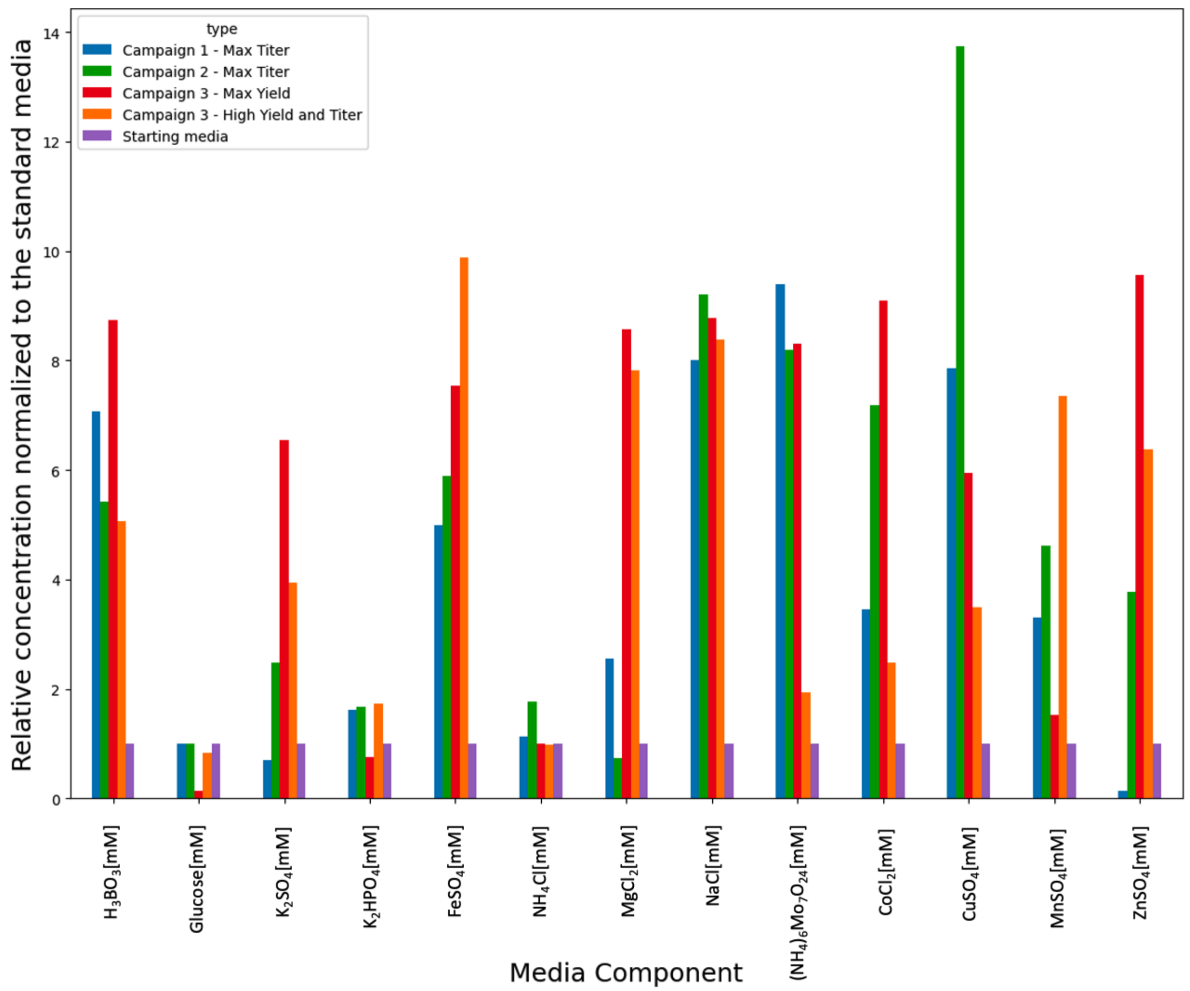

Figure S6. The optimal media from all three campaigns all converged to similar final media designs. Regardless of optimization objective and user/campaign, the majority of the most important media components (NaCl, $FeSO_4$, K_2HPO_4 and NH_4Cl) end up having relatively similar concentrations. K_2SO_4 shows different results, however. The orange bars (Campaign 3 - High Yield and Titer) correspond to a media design that produced both high yield and high titer in campaign 3 (The shaded square area in **Fig. 6**).

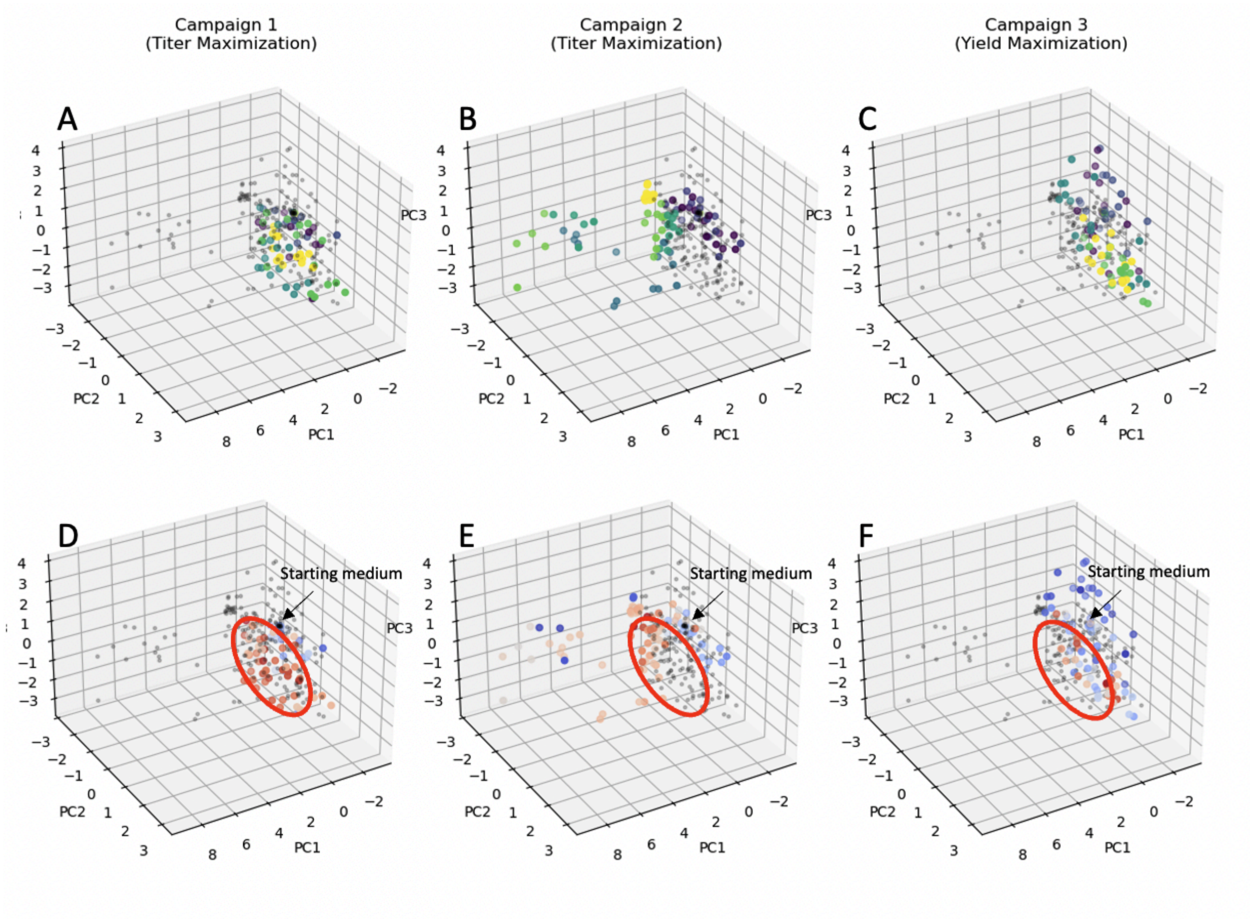

Figure S7: The first 2 principal components capture most of the variance in all three campaigns. We used principal component analysis (PCA) to identify the most important dimensions along which most of the variance is explained. We can see that the highest performing media fall within the same region, circled red, both for titer maximization (Campaign 1, panels A, D and Campaign 2, panels B, E) and yield maximization (Campaign 3, panels C, F). Panels A, B, and C show compositions colored by DBTL cycle, while Panels D, E, and F show compositions colored by flaviolin production levels. The first two principal components describe 43% of the total variance (Fig. 5), and the third principal component explains 9.4% of the total variance, adding up to 52%.

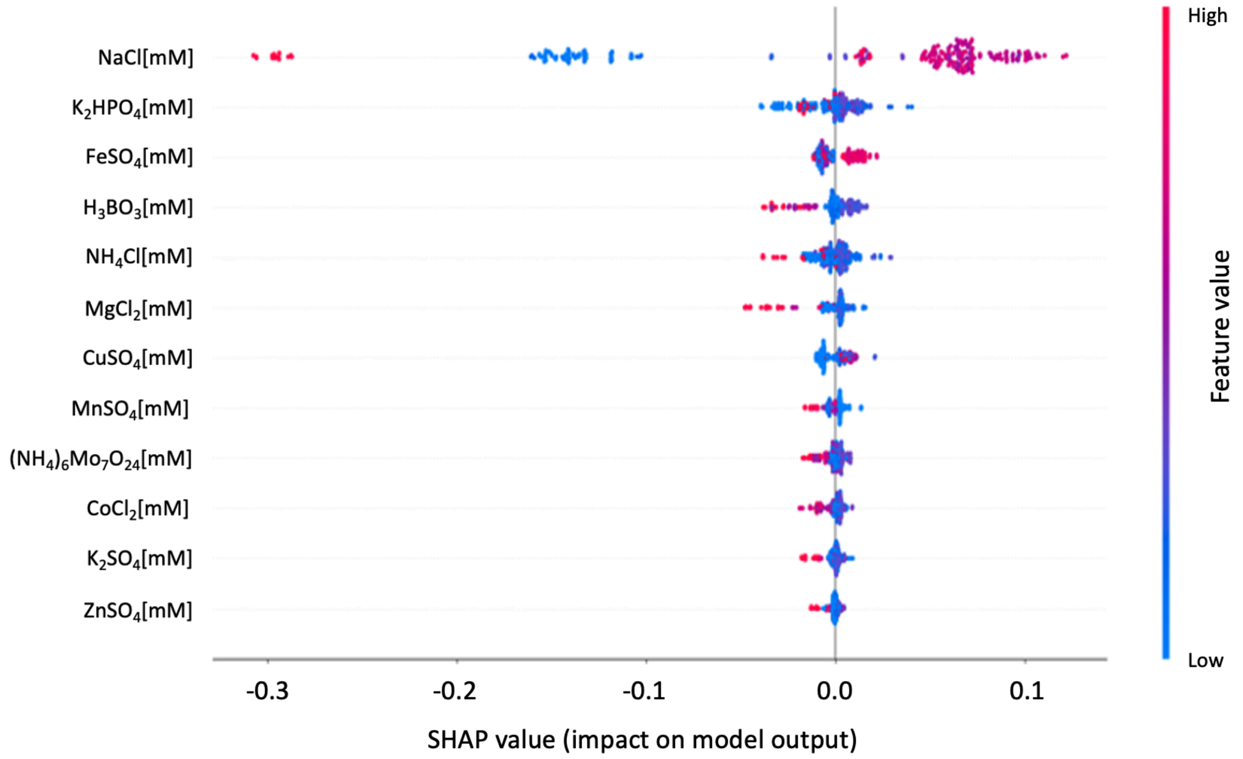

Figure S8: Feature importance for campaign 2 shares most of the top drivers with campaign 1. As in campaign 1, NaCl concentration is the main driver of production improvement. There are six top drivers that include the main five top drivers from campaign 1 (Fig. 7), except for K_2SO_4. These six top drivers also include H_3BO_3 (11th position for campaign 1).

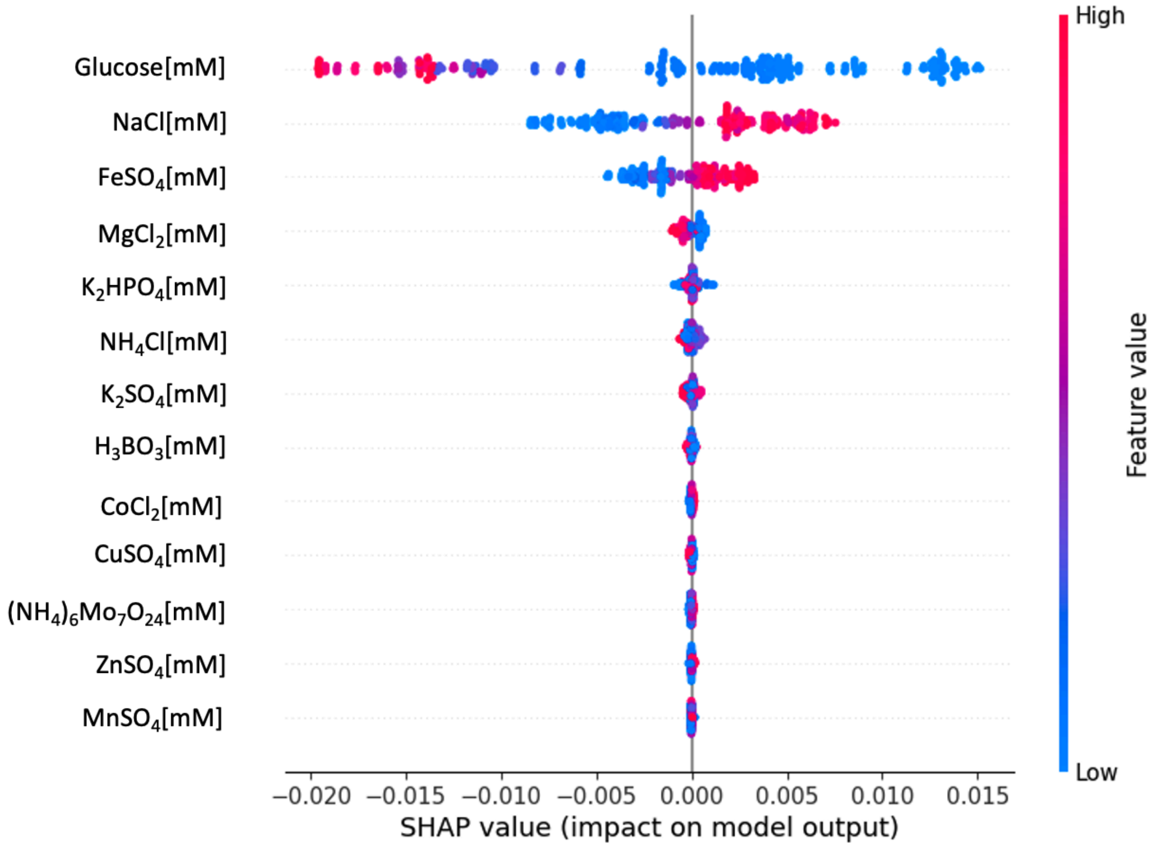

Figure S9: Feature importance analysis for campaign 3 shares all the top drivers with campaign 1 and 2, except for glucose. Glucose (fixed in campaigns 1 and 2) is, unsurprisingly, the main driver, but NaCl closely follows. The other factor in the top three most important drivers is $FeSO_4$, present in the top drivers in campaign 1 and 2.

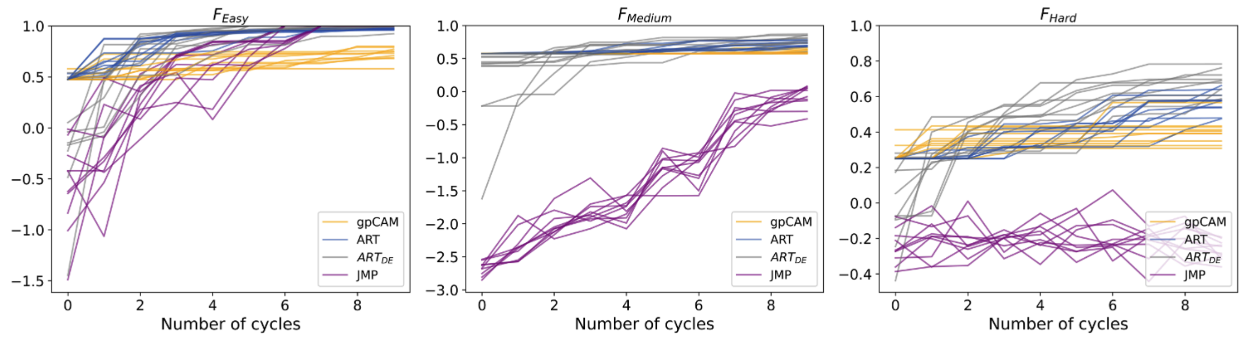

Figure S10: Active learning shows a characteristic "bursty" behavior in which response improvements come in bursts rather than as a smooth improvement when more data is added. This figure is the same as **Fig. 8**, but the ten different processes were not averaged, showing that process improvement comes in bursts.

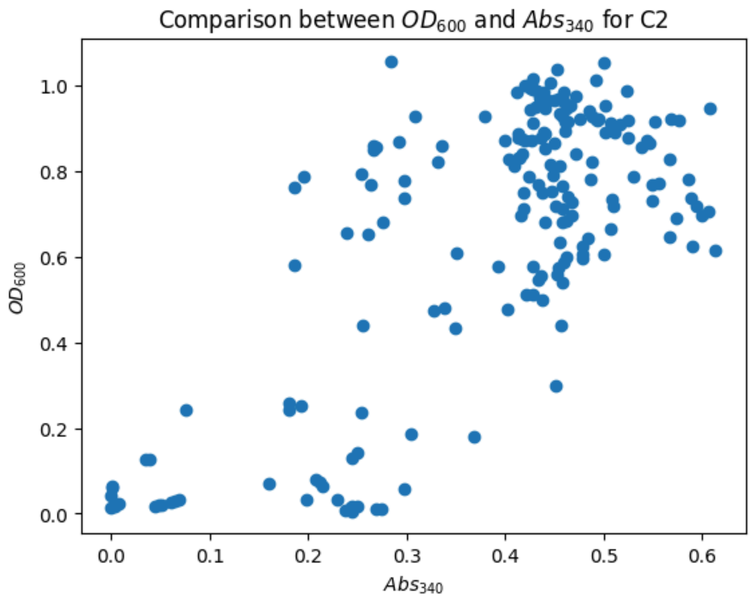

Figure S11: Cellular growth (OD_{600}) is not unduly diminished by high flaviolin production (Abs_{340}). This is exemplary data from campaign 2 (C2). Data from Campaigns 1 and 3 support a similar conclusion.

Table S1: Components of optimal media concentrations for all three campaigns, compared with the starting/control media (MOPS minimal media), an intermediate media composition for campaign C1 (DBTL 4), and a popular media for *P. putida*: M9 media. M9 media is a defined minimal media routinely used for *P. putida*, and is shown for comparison purposes.

**Component**	**C1 Optimal Media Concentration[mM] - Max Titer**	**C2 Optimal Media Concentration[mM] - Max Titer**	**C3 Optimal Media Concentration[mM] - Max Yield**	**Initial MOPS Minimal Media Concentration[mM]**	**Intermediate media composition from C1-DBTL4 [mM]**	**M9 (1) media Concentration[mM]**
H_3BO_3	0.28	0.022	0.035	0.004	0.038	0.000043
K_2SO_4	0.20	0.71	1.90	0.29	0.8	n/a
K_2HPO_4	2.14	2.21	0.99	1.32	1.42	~40mM equivalent in 21mM Na_2HPO_4 and 11.3 KH_2PO_4
$FeSO_4$	0.050	0.059	0.075	0.01	0.08	n/a
NH_4Cl	10.68	16.81	9.46	9.52	13.71	9.35
$MgCl_2$	1.33	0.38	4.45	0.52	1.46	n/a
NaCl	400.02	460.10	438.65	50	323.9	4.28
$(NH_4)_2Mo_7O_2$	0.00028	0.00025	0.00025	0.00003	0.00030	0.0000016
$CoCl_2$	0.0010	0.0022	0.027	0.0003	0.0021	n/a
$CuSO_4$	0.00079	0.0014	0.00060	0.0001	0.001	0.00000031
$MnSO_4$	0.0026	0.0037	0.0012	0.0008	0.005	n/a
$ZnSO_4$	0.000014	0.00038	0.00096	0.0001	0.0008	0.00000077
MOPS	40	40	40	40	40	n/a
Tricine	4	4	4	4	4	n/a
Glucose	20	20	2.62	20	20	22

Table S2: Recommendation strategy for each DBTL cycle for each campaign. The objective function used for sampling the space and the use of α is described in Eq. 1.

	Cycle	# of exploration recommendations	α exploration	# of exploitation recommendations	α exploitation
Campaign 1 - Titer maximization	DBTL 3	12	0.9	3	0
	DBTL 4	8	1	7	0
	DBTL 5	5	1	10	0
Campaign 2 - Titer maximization	DBTL 3	7	1	8	0
	DBTL 4	5	1	10	0
	DBTL 5	5	1	10	0
	DBTL 6	2	1	13	0
Campaign 3 - Yield maximization	DBTL 3	10	1	5	0
	DBTL 4	7	1	8	0
	DBTL 5	3	1	12	0

References

1. Soma Y, Tominaga S, Tokito K, Imado Y, Naka K, Hanai T, et al. Trace impurities in sodium phosphate influences the physiological activity of Escherichia coli in M9 minimal medium. Sci Rep. 2023 Oct 13;13(1):17396.
