# Supplementary material for: Machine learning-led semi-automated medium optimization reveals salt as key for flaviolin production in Pseudomonas putida
Source: Commun Biol. 2025 Apr 18;8:630. doi: 10.1038/s42003-025-08039-2 (PMC12008372; doi:10.1038/s42003-025-08039-2)
Supplement: Supplementary file 3 — Reporting summary [file 42003_2025_8039_MOESM3_ESM.pdf]

nature portfolio

Corresponding author(s): Hector Garcia-Martin

Last updated by author(s): Mar 27, 2025

Reporting Summary

Nature Portfolio wishes to improve the reproducibility of the work that we publish. This form provides structure for consistency and transparency in reporting. For further information on Nature Portfolio policies, see our Editorial Policies and the Editorial Policy Checklist.

Statistics

For all statistical analyses, confirm that the following items are present in the figure legend, table legend, main text, or Methods section.

n/a	Confirmed	
☐	☒	The exact sample size (*n*) for each experimental group/condition, given as a discrete number and unit of measurement
☐	☒	A statement on whether measurements were taken from distinct samples or whether the same sample was measured repeatedly
☐	☒	The statistical test(s) used AND whether they are one- or two-sided<br>*Only common tests should be described solely by name; describe more complex techniques in the Methods section.*
☐	☒	A description of all covariates tested
☐	☒	A description of any assumptions or corrections, such as tests of normality and adjustment for multiple comparisons
☐	☒	A full description of the statistical parameters including central tendency (e.g. means) or other basic estimates (e.g. regression coefficient) AND variation (e.g. standard deviation) or associated estimates of uncertainty (e.g. confidence intervals)
☒	☐	For null hypothesis testing, the test statistic (e.g. *F*, *t*, *r*) with confidence intervals, effect sizes, degrees of freedom and *P* value noted<br>*Give P values as exact values whenever suitable.*
☒	☐	For Bayesian analysis, information on the choice of priors and Markov chain Monte Carlo settings
☒	☐	For hierarchical and complex designs, identification of the appropriate level for tests and full reporting of outcomes
☒	☐	Estimates of effect sizes (e.g. Cohen's *d*, Pearson's *r*), indicating how they were calculated

Our web collection on statistics for biologists contains articles on many of the points above.

Software and code

Policy information about availability of computer code

Data collection	The media compiler was used to generate instructions for the Biomek NX-S8. This is available through Zenodo
Data analysis	JMP 17 and the Automated recommendation tool were used in this study for Data Analysis, as described in the manuscript. Code used has been made available through Zenodo

For manuscripts utilizing custom algorithms or software that are central to the research but not yet described in published literature, software must be made available to editors and reviewers. We strongly encourage code deposition in a community repository (e.g. GitHub). See the Nature Portfolio guidelines for submitting code & software for further information.

Data

Policy information about availability of data

All manuscripts must include a data availability statement. This statement should provide the following information, where applicable:
- Accession codes, unique identifiers, or web links for publicly available datasets
- A description of any restrictions on data availability
- For clinical datasets or third party data, please ensure that the statement adheres to our policy

The experimental data can be found in the corresponding repository for each study named “DBTL1-5.csv” for C1 and C3 or “DBTL1-6.csv” for C2. The data can also be accessed through the JBEI instance of the Experiment Data Depot (44) (public-edd.jbei.org)

Research involving human participants, their data, or biological material

Policy information about studies with human participants or human data. See also policy information about sex, gender (identity/presentation), and sexual orientation and race, ethnicity and racism.

Reporting on sex and gender	n/a
Reporting on race, ethnicity, or other socially relevant groupings	n/a
Population characteristics	n/a
Recruitment	n/a
Ethics oversight	n/a

Note that full information on the approval of the study protocol must also be provided in the manuscript.

Field-specific reporting

Please select the one below that is the best fit for your research. If you are not sure, read the appropriate sections before making your selection.

☒ Life sciences ☐ Behavioural & social sciences ☐ Ecological, evolutionary & environmental sciences

For a reference copy of the document with all sections, see nature.com/documents/nr-reporting-summary-flat.pdf

Life sciences study design

All studies must disclose on these points even when the disclosure is negative.

Sample size	3-4 biological replicates were used for each media composition
Data exclusions	n each cycle, we identified two types of outliers, likely stemming from errors by the liquid handler: 1) cases where one of three replicates did not produce any biomass or flaviolin, possibly due to an error when inoculating the well, and 2) cases where one triplicate had significantly higher Abs340 than the others, possibly due to transferring cells along with the supernatant. In both of these cases these media designs were removed from the pipeline and were not used to train ART.
Replication	We used a control media design in all DBTL cycles. We also performed the same experiments with different users, using different stock solutions and more than a year apart to ensure reproducibility of our experimental pipeline. We also re-run the whole active learning process multiple times and got to the same results.
Randomization	The active learning process we implemented is inherently stochastic, so there was randomization in our methods.
Blinding	All of the data collection was performed using a our semi-automated experimental pipeline with the minimal amount of experimenter intervention. The data analysis was performed using the same jupyter notebooks for each DBTL cycle, with minimal user intervention and the media recommendations were tested in the next DBTL cycle.

Reporting for specific materials, systems and methods

We require information from authors about some types of materials, experimental systems and methods used in many studies. Here, indicate whether each material, system or method listed is relevant to your study. If you are not sure if a list item applies to your research, read the appropriate section before selecting a response.

Materials & experimental systems

n/a	Involved in the study	
☒	☐	Antibodies
☒	☐	Eukaryotic cell lines
☒	☐	Palaeontology and archaeology
☒	☐	Animals and other organisms
☒	☐	Clinical data
☒	☐	Dual use research of concern
☒	☐	Plants

Methods

n/a	Involved in the study	
☒	☐	ChIP-seq
☒	☐	Flow cytometry
☒	☐	MRI-based neuroimaging

Plants

Seed stocks	n/a
Novel plant genotypes	n/a
Authentication	n/a
